# Potential Uses for Cord Blood Mesenchymal Stem Cells

**Published:** 2013-11-20

**Authors:** Morteza Zarrabi, Seyed Hadi Mousavi, Saeid Abroun, Bahareh Sadeghi

**Affiliations:** 1 Royan Stem Cell Technology, Cord Blood Bank, Tehran, Iran; 2 Department of Hematology, School of Medical Sciences, Tarbiat Modares University, Tehran, Iran

**Keywords:** Cord Blood, Mesenchymal Stem Cells, Transplantation

## Abstract

Stem cell therapy is a powerful technique for the treatment of a number of diseases. Stem cells are derived from different tissue sources, the most important of which are the bone marrow (BM), umbilical cord (UC) blood and liver. Human UC mesenchymal stem cells (hUC-MSCs) are multipotent, non-hematopoietic stem cells that have the ability to self-renew and differentiate into other cells and tissues such as osteoblasts, adipocytes and chondroblasts. In a number of reports, human and mouse models of disease have hUC-MSCs treatments. In this article, we review studies that pertain to the use of hUC-MSCs as treatment for diseases.

## Introduction

Now a days, regenerative medicine in stem cell filed widely attractive by scientists. Stem cell therapy is a potential method for treatment of some disorders (1). Sources for stem cells vary, each of which have uses for certain diseases (2-4). Mesenchymal stem cells (MSCs) are one source for stem cells that are multipotent, non-hematopoietic and have the capability for self-renewal and differentiation (5,6). MSCs can be isolated from bone marrow (BM), cord blood, placenta, adipose tissue and liver (2,3,7,8)

**Fig 1:**
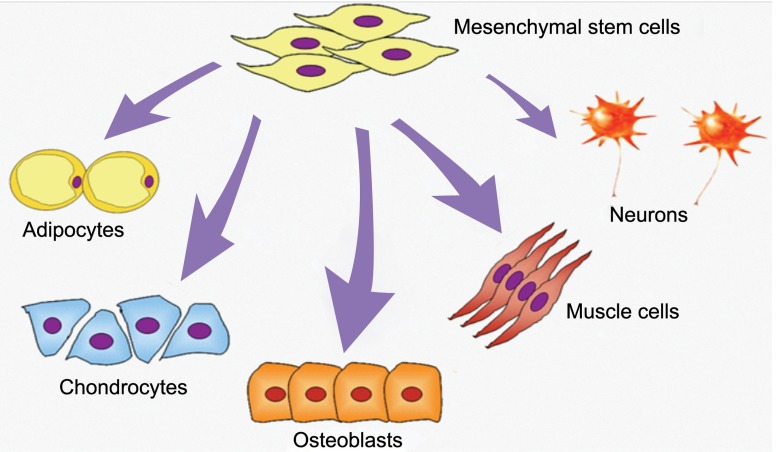
Differentiation potential of MSCs (26).

According to the International Society for Cellular Therapy (ISCT), MSCs are distinguished by their ability: 1. adhere to plastic (when maintained in standard tissue culture flasks these cells should adhere to the bottom of the flask); 2. express specific surface antigens (>95% of these cells, express CD73, CD90 and CD105 and are negative for CD45, CD34, CD14 or CD11b, CD79a or CD19 and HLA-II); and 3. under standard differentiating conditions *in vitro* they have the capability to differentiate into osteoblasts, adipocytes and chondroblasts (Table 1,Fig 1) (9). In addition, MSCs should have the capability to differentiate into endothelial, epithelial, hepatocytes and neural cells (10-14). Other properties of MSCs include ease of separation, immunomodulatory effects, migratory behavior and no ethical limitations (15).

**Table 1:** Summary of criteria for the identification of MSCs (9)


**1**	**Adherence to plastic under standard culture conditions**		
**2**	**Phenotype**	Positive (≥95% +)	Negative (≤2% +)
		CD105	CD45
		CD73	CD34
		CD90	CD14 or CD11b
			CD79α or CD19
			HLA-DR
**3**	***In vitro* differentiation: into osteoblasts, adipocytes, and chondroblasts**		


In addition to BM, the human umbilical cord (UC) is an alternative source for MSCs (16,17). UC-MSCs for stem cell therapy have advantages over BM-MSCs because they are ease available, collection from the donor is neither invasive nor painful, and there are no ethical considerations (18). UC-MSCs are more primitive than BMMSCs and have the capability to differentiate into different cells (19-22). In addition to the previously mentioned CD markers, they also express CD29, CD44, CD49b, CD58, CD166 and HLA-I. They are negative for CD3, CD7, CD33, CD40, CD49d, CD80, CD86, CD117, and CD133 (23-25).

The current review discusses pre-clinical and clinical human umbilical cord mesenchymal stem cell (hUC-MSCs) application in the treatment of some disorders.

### Regenerative medicine

Regenerative medicine is the process by which human cells and tissues are used to restore normal function. In this field, MSCs have the capability to differentiate into multiple cell lineages such as osteoblasts, chondrocytes and adipocytes.

### Neurological disorders

#### Cerebral ischemia

Cerebral ischemia, also known as brain ischemia, results from a lack of blood flow to the brain that leads to cerebral hypoxia and results in neurological injury. This condition results in major complications such as impairments in vision, physical movement, and speech. If the appropriate diagnosis and treatment are not performed, symptoms may be permanent and life-threatening. Neurological recovery following stem cell transplantation in animal model with ischemia has shown the safety and effectiveness of this type of transplantation (27). Several studies have reported in general that hUC-MSC transplantation into the cortex of an occluded middle cerebral artery can result in the successful in recovery of neurological function in a mice model (28). In this process have been shown that hUC-MSCs migrate to the ischemic area and differentiate into neurons, glial and other different types of neural cells. There appears to be increased cortical blood flow in the ischemic zone because of angiogenesis by hUC-MSCs. Koh et al. (29) have shown recovery of neurobehavioral function and decreased infarct volume after transplantation by hUCMSCs. This group of researchers has shown that the neuroprotective effect of hUC-MSCs caused an improvement in behavioral function. According to other study hUC-MSCs transplantation significantly reduced the injury mass and deficiency of neurological function due to angiogenesis in a rat model of ischemic cerebral injury (30).

#### Spinal cord injury

Spinal cord injury (SCI) that arises from damage or trauma to the spinal cord because of loss or disturbed function results in reduced mobility (31). Yang et al. (32) have recently published impressive data that hUC-MSC transplantation can be a potential strategy for the promotion of corticospinal fiber regeneration and improvement in locomotor function following spinal cord transection into the lesion area of SCI rats compared with a control group. The results showed increased numbers of regenerated axons in the corticospinal area of the zone around the SCI lesion. Migration of hUC-MSCs from the implantation zone was observed. This group of researchers hypothesized that the release of cytokines and grow factors from stem cells was the key mechanism for corticospinal fiber progression.

#### Parkinson disease

Parkinson disease (PD) is a neurodegenerative disorder defined by progressive loss of striatal dopaminergic function (33). Fu et al. (34) have reported in a PD rat model with an infusion of hUC-MSC into the striatum resulted in a partial improvement the lesion-induced amphetamine-evoked rotation. In another study, Weiss et al. (35) found the same results in a hemiparkinsonian rat model.

#### Alzheimer’s disease

Alzheimer’s disease (AD) is a neurodegenerative disorder characterized by loss of neurons and synapses in the cerebral cortex, dementia and histopathological symptoms such as senile plaques and neurofibrillary tangles (36). A deposit of amyloid-β peptide (Aβ) in the brain is the fundamental cause of this disease (37). In addition, oxidative stress and inflammatory effects may have a pathological role in AD. AD is progressive and to date, incurable (38-40). There are some evidences for the efficacy of cell therapy in AD. Lee et al. (41) have shown that use of hUC-MSCs in cell culture medium and an AD mouse model can reduce and ameliorate disease symptoms. This group has reported that co-culture of hUC-MSCs with hippocampal neurons resulted in a significant reduction in apoptosis compared with the control group. Also they have shown that injection of Aβ in the brain of a mice model that transplantanted hUC-MSCs led to diminished oxidative stress and glial activation compared with the control group. Stephan et al. (42) and Tsai et al. (43) reported that hUC-MSCT suppressed activated astrocytes and microglia activation which increased in the test group. According to this data, they have suggested that hUC-MSCT cells may have a neuroprotective role. Learning and memory can be improved by administration of hUC-MSCT in an acute AD mice model.

#### Support of hematopoiesis

MSCs have the potential capacity to support hematopoietic stem cell growth both *in vivo* and *in vitro* (44,45). The main mechanism of hematopoiesis involves secretion of a number of major hematopoietic cytokines such as IL- 6, IL-7, IL-8, IL-11, IL-14, IL-15, M-CSF and SCF (20). In a study by Bakhshi et al. (46), it was shown that hUC-MSCs could support the growth of CD34+ cord blood cells as determined by long-term culture initiation cell culture (LTC-IC). In another study, researchers have shown that UC-MSC compared with BM had the capabilityto produce hematopoietic growth factors such as IL-6, IL-8, IL-11, G-CSF, GMCSF and LIF (25). Researchers show that hUCMSC increases homing and migration of UCB CD34+ cells to the BM and spleen. According to evidence, there is low efficacy of UC-MSC hematopoietic support capacity compared with BM however UC-MSCs have the ability to support long-term hematopoiesis *in vitro* (47).

#### Autoimmune diseases

Immunomodulatory effects of MSC have a critical role in the mechanism of autoimmune diseases. MSCs express low levels of HLA-I, but do not express HLA-II co-stimulatory molecules such as CD80, CD86 and CD40. In addition, these cells suppress activated T-cell proliferation and differentiation (48).

#### Type 1 diabetes mellitus

Type 1 diabetes mellitus (T1DM) or juvenile diabetes is an autoimmune disease of insulinproducing beta cells of the pancreas (49,50). Although this disease can occur in any age group, it is mostly diagnosed in children and young adults. Common methods for controlling T1DM are insulin injections and self-monitoring blood glucose levels. Islet transplantation is the most efficient medication for patients diagnosed with insulin-dependent diabetes. Some studies have shown the application of different kinds of stem cells (embryonic, pancreatic, hepatic, BM and UC blood) in insulin production (51-56). Researchers have produced islet-like cells *in vitro* from hUC-MSCs which were then infused into the livers of diabetic rats. After transplantation, these cells secreted human C-peptide and in response to physiological glucose levels, released human insulin.

Advantages such as availability, large donor pool, non-invasive to the donor and low risk of rejection exist for using transplanted hUC-MSCs (47,57). Limitations exist for UC, BM and ESC such as the low amount of UC, limited expansion and differentiation of BM, and ethical issues with the use of ESCs. Therefore transplantation of hUC-MSCs is the most efficient method for treatingT1DM (58).

#### Systemic lupus erythematosus

Systemic lupus erythematosus (SLE) is a systemic autoimmune disease that can affect any part of the body. SLE occurs more often in women than men (59). This disease may be the result of an attack by the immune system on cells and tissues of the body that occurs by producing antibodies against itself. SLE most often causes damage to the heart, joints, skin, lungs, blood vessels, liver, kidneys, and nervous system. In a research study, Sun et al. (60) choses patients with severe SLE refractory to conventional immunosuppressive or immunomodulatory therapy (glucocorticoids, cyclophosphamide and mycophenolate mofetil) to receive hUC-MSCs. Following transplantation, patients were evaluated according to the SLE disease activity index (SLEDAI) which assessed anti-nuclear antibody (ANA), antidouble stranded DNA (anti-dsDNA) antibody, levels of serum complement C3, C4 and albumin, and renal function (60). After a period of one month, SLEDAI scores significantly decreased in all 16 patients. The results of this study stated that additional decreases in SLEDAI were observed after three and six months. At three months following hUC-MSC transplantation, levels for 24- hour proteinuria, serum creatinine and urea nitrogen were measured for assessment of renal function. All of these parameters decreased and additionally, serum albumin returned to normal levels. As an indicator of immune system response, the researchers evaluated Treg cells (CD4+ FoxP3+ T cells). The results showed increased numbers of Treg cells and the ratio of Th1 and Th2 reestablished after UC-MSCT. It postulated that an immune mechanism played a key role in the response to a successful engraftment (60).

#### Rheumatoid arthritis

Rheumatoid arthritis (RA) is a chronic, systemic inflammatory disorder that involves 1% of the world’s population and most frequently occurs in women. This disease affects numerous tissues and organs, but the synovial joints are the major target (61). The most common adverse effects of RA are destruction of articular cartilage and ankylosis of the joints (62). Proinflammatory cytokines such as TNF-α, IL- 6, IL-1β and IL-17 have an important role in pathogenesis of RA (63). There is no known cure, however treatment can lessen symptoms. New therapeutic agents such as TNF-α and B cell depleting therapy exist, however they are expensive and not usually used (64). Today, UC-MSCT is a new therapeutic method for treatment of RA. Liu et al. (63) by co-culturing UC-MSCs with synovial tissue harvested from patients with RA, have shown that UC-MSC inhibited fibroblast-like synoviocytes (FLSs). FLSs, are resident cells of synovial joints that have a crucial role in inflammation and joint destruction. UC-MSCs suppressed the invasive behavior, MMP-9 expression and inflammatory response of FLSs. In addition, Treg cells that play an important role in maintenance of selfimmune tolerance in RA significantly increased. Assessment of UC-MSC on collagen-induced arthritis (CIA) in a mouse model explored that these cells prevented tissue damage, reduced inflammatory responses and Re-established the ratio between Th1 and Th2 cells. The results showed that, UC-MSCs significantly improved CIA in mice.

#### Wound healing

Delay and lack of skin reconstitution, infection, decreased circulation, low nutrition and low numbers of stem cells are common problems in persons with deep wounds (65). In a study of a mice model with a full skin defect, injection of hUC-MSCs in the wound site has shown significantly enhanced wound healing with a much thicker newly formed epidermis layer in the experimental group compared with the control group (66). A significant increase in the amount of cells was observed in the regenerated skin tissue with improved dermal ridges. In the experimental group, folliculus pili and other appendixes had an important role in repairing skin tissue which was not observed in the control group. UC-MSCT caused more rapid, enhanced and improved wound healing.

## Conclusion

Various methods exist as treatment for different diseases. One of the newest methods in regenerative medicine is stem cell therapy. Because stem cells have special advantages such as the ability to self-renew and differentiate into different cells and tissues, these cells are appropriate sources as treatment for several disorders. In addition to BM, the UC is a rich source of MSCs. MSCs are a source of stem cells that have potential power to improve responses to diseases such as T1DM, cerebral ischemia, SCI, PD, SLE and RA. There are potential advantages such as the profound immunomodulatory effects in the application of MSCs as treatment for different disorders.

## References

[1] Perdikogianni C, Dimitriou H, Stiakaki E, Martimianaki G, Kalmanti M (2008). Could cord blood be a source of mesenchymal stromal cells for clinical use?. Cytotherapy.

[2] Erices A, Conget P, Minguell JJ (2000). Mesenchymal progenitor cells in human umbilical cord blood. Br J Haematol.

[3] In 't Anker PS, Scherjon SA, Kleijburg-van der Keur C, de Groot-Swings GM, Claas FH, Fibbe WE (2004). Isolation of mesenchymal stem cells of fetal or maternal origin from human placenta. Stem Cells.

[4] Panepucci RA, Siufi JL, Silva WA Jr, Proto-Siquiera R, Neder L, Orellana M (2004). Comparison of gene expression of umbilical cord vein and bone marrow-derived mesenchymal stem cells. Stem Cells.

[5] Jiang Y, Jahagirdar BN, Reinhardt RL, Schwartz RE, Keene CD, Ortiz-Gonzalez XR (2002). Pluripotency of mesenchymal stem cells derived from adult marrow. Nature.

[6] Mareschi K, Ferrero I, Rustichelli D, Aschero S, Gammaitoni L, Aglietta M (2006). Expansion of mesenchymal stem cells isolated from pediatric and adult donor bone marrow. J Cell Biochem.

[7] Campagnoli C, Roberts IA, Kumar S, Bennett PR, Bellantuono I, Fisk NM (2001). Identification of mesenchymal stem/progenitor cells in human first-trimester fetal blood, liver, and bonemarrow. Blood.

[8] Wang HS, Hung SC, Peng ST, Huang CC, Wei HM, Guo YJ (2004). Mesenchymal stem cells in the Wharton’s jelly of the human umbilical cord. Stem Cells.

[9] Dominici M, Le Blanc K, Mueller I, Slaper-Cortenbach I, Marini F, Krause D (2006). Minimal crite ria for defining multipotent mesenchymal stromal cells.The International Society for Cellular Therapy position statement. Cytotherapy.

[10] Woodbury D, Schwarz EJ, Prockop DJ, Black IB (2000). Adult rat and human bone marrow stromal cells differentiate into neurons. J Neurosci Res.

[11] Chagraoui J, Lepage-Noll A, Anjo A, Uzan G, Charbord P (2003). Fetal liver stroma consists of cells in epithelial-to-mesenchymal transition. Blood.

[12] Spees JL, Olson SD, Ylostalo J, Lynch PJ, Smith J, Perry A (2003). Differentiation, cell fusion, and nuclear fusion during ex vivo repair of epithelium by human adult stem cells from bone marrow stroma. Proc Natl Acad Sci USA.

[13] Oswald J, Boxberger S, Jørgensen B, Feldmann S, Ehninger G, Bornhauser M (2004). Mesenchymal stem cells can be differentiated into endothelial cells in vitro. Stem Cells.

[14] Ma Y, Xu Y, Xiao Z, Yang W, Zhang C, Song E (2006). Reconstruction of chemically burned rat corneal surface by bone marrow-derived human mesenchymal stemcells. Stem Cells.

[15] Brooke G, Cook M, Blair C, Han R, Heazlewood C, Jones B (2007). Therapeutic applications of mesenchymal stromal cells. Semin Cell Dev Biol.

[16] Flynn A, Barry F, O’Brien T (2007). UC blood-derived mesenchymal stromal cells: an overview. Cytotherapy.

[17] Secco M, Zucconi E, Vieira NM, Fogaça LL, Cerqueira A, Carvalho MD (2008). Multipotent stem cells from umbilical cord: cord is richer than blood. Stem Cells.

[18] Wu LF, Wang NN, Liu YS, Wei X (2009). 20.Differentiation of Wharton’s jelly primitive stromal cells into insulin-producing cells in comparison with bonemarrow mesenchymal stem cells. Tissue Eng Part A.

[19] Sarugaser R, Lickorish D, Baksh D, Hosseini MM, Davies JE (2005). Human umbilical cord perivascular (HUCPV) cells: a source of mesenchymal progenitors. Stem Cells.

[20] Lu LL, Liu YJ, Yang SG, Zhao QJ, Wang X, Gong W (2006). Isolation and characterization of human umbilical cord mesenchymal stem cells with hematopoiesis- supportive function and other potentials. Haematologica.

[21] Can A, Karahuseyinoglu S (2007). Concise review: human umbilical cord stroma with regard to the source of fetus-derived stem cells. Stem Cells.

[22] Wu KH, Zhou B, Lu SH, Feng B, Yang SG, Du WT (2007). In vitro and *in vivo* differentiation of human umbilical cord derived stem cells into endothelial cells. J Cell Biochem.

[23] Bieback K, Klüter H (2007). Mesenchymal stromal cells from umbilical cord blood. Curr Stem Cell Res Ther.

[24] Malgieri A, Kantzari E, Patrizi MP, Gambardella S (2010). Bone marrow and umbilical cord blood human mesenchymal stem cells: state of the art. Int J Clin Exp Med.

[25] Friedman R, Betancur M, Boissel L, Tuncer H, Cetrulo C, Klingemann H (2007). Umbilical cord mesenchymal stem cells: adjuvants for human cell transplantation. Biol Blood Marrow Transplant.

[26] Meregalli M, Farini A, Torrente Y (2011). Mesenchymal stem cells as muscle reservoir. J Stem Cell Res Ther.

[27] Hess DC, Borlongan CV (2008). Cell-based therapy in ischemic stroke. Expert Rev Neurother.

[28] Ding DC, Shyu WC, Chiang MF, Lin SZ, Chang YC, Wang HJ (2007). Enhancement of neuroplasticity through upregulation of beta1-integrin in human umbilical cord-derived stromal cell implanted stroke model. Neurobiol Dis.

[29] Koh SH, Kim KS, Choi MR, Jung KH, Park KS, Chai YG (2008). Implantation of human umbilical cord-derived mesenchymal stem cells as a neuroprotective therapy forischemic stroke in rats. Brain Res.

[30] Liao W, Zhong J, Yu J, Xie J, Liu Y, Du L (2009). Therapeutic benefit of human umbilical cord derived mesenchymal stromal cells in intracerebral hemorrhagerat: implications of anti-inflammation and angiogenesis. Cell Physiol Biochem.

[31] Coutts M, Keirstead HS (2008). Stem cells for the treatment of spinal cord injury. Exp Neurol.

[32] Yang CC, Shih YH, Ko MH, Hsu SY, Cheng H, Fu YS (2008). Transplantation of human umbilical mesenchymal stem cells from Wharton’s jelly after complete transection of the rat spinal cord. PLoS One.

[33] Yasuhara T, Date I (2007). Intracerebral transplantation of genetically engineered cells for Parkinson’s disease: toward clinical application. Cell Transplant.

[34] Fu YS, Cheng YC, Lin MY, Cheng H, Chu PM, Chou SC (2006). Conversion of human umbilical cord mesenchymal stem cells in Wharton’s jelly to dopaminergic neurons in vitro: potential therapeutic application for Parkinsonism. Stem Cells.

[35] Weiss ML, Medicetty S, Bledsoe AR, Rachakatla RS, Choi M, Merchav S (2006). Human umbilical cord matrix stem cells: preliminary characterization and effect of transplantation in a rodentmodel of Parkinson’s disease. Stem Cells.

[36] Zhu X, Raina AK, Perry G, Smith MA (2006). Apoptosis in Alzheimer disease: a mathematical improbability. Curr Alzheimer Res.

[37] Madeira A, Pommet JM, Prochiantz A, Allinquant B (2005). SET protein (TAF1beta, I2PP2A) is involved in neuronal apoptosis induced by an amyloid precursor proteincytoplasmic subdomain. FASEB J.

[38] Wyss-Coray T, Mucke L (2002). Inflammation in neurodegenerative disease--a double-edged sword. Neuron.

[39] Behl C (2005). Oxidative stress in Alzheimer’s disease: implications for prevention and therapy. Subcell Biochem.

[40] Onyango IG, Khan SM (2006). Oxidative stress, mitochondrial dysfunction, and stress signaling in Alzheimer’s disease. Curr Alzheimer Res.

[41] Lee HJ, Lee JK, Lee H, Shin JW, Carter JE, Sakamoto T (2010). The therapeutic potential of human umbilical cord blood-derived mesenchymal stem cells in Alzheimer’s disease. Neurosci Lett.

[42] Stéphan A, Laroche S, Davis S (2001). Generation of aggregated beta-amyloid in the rat hippocampus impairs synaptic transmission andplasticity and causes memory deficits. J Neurosci.

[43] Tsai KJ, Tsai YC, Shen CK (2007). G-CSF rescues the memory impairment of animal models of Alzheimer’s disease. J Exp Med.

[44] Li N, Feugier P, Serrurrier B, Latger-Cannard V, Lesesve JF, Stoltz JF (2007). Human mesenchymal stem cells improve ex vivo expansion of adult human CD34+ peripheral bloodprogenitor cells and decrease their allostimulatory capacity. Exp Hematol.

[45] Kim DW, Chung YJ, Kim TG, Kim YL, Oh IH (2004). Cotransplantation of third-party mesenchymal stromal cells can alleviate single-donor predominanceand increase engraftment from double cord transplantation. Blood.

[46] Bakhshi T, Zabriskie RC, Bodie S, Kidd S, Ramin S, Paganessi LA (2008). Mesenchymal stem cells from the Wharton’s jelly of umbilical cord segments provide stromal supportfor the maintenance of cord blood hematopoietic stem cells during long-term ex vivo culture. Transfusion.

[47] Fan CG, Zhang QJ, Zhou JR (2011). Therapeutic potentials of mesenchymal stem cells derived from human umbilical cord. Stem Cell Rev.

[48] Kim JY, Jeon HB, Yang YS, Oh W, Chang JW (2010). Application of human umbilical cord blood-derived mesenchymal stem cells in disease models. World J Stem Cells.

[49] Tydén G, Reinholt FP, Sundkvist G, Bolinder J (1996). Recurrence of autoimmune diabetes mellitus in recipients of cadaveric pancreatic grafts. N Engl J Med.

[50] Atkinson MA, Eisenbarth GS (2001). Type 1 diabetes: new perspectives on disease pathogenesis and treatment. Lancet.

[51] Ramiya VK, Maraist M, Arfors KE, Schatz DA, Peck AB, Cornelius JG (2000). Reversal of insulin-dependent diabetes using islets generated in vitro from pancreatic stem cells. Nat Med.

[52] Lumelsky N, Blondel O, Laeng P, Velasco I, Ravin R, McKay R (2001). Differentiation of embryonic stem cells to insulin-secreting structures similar to pancreatic islets. Science.

[53] Yang L, Li S, Hatch H, Ahrens K, Cornelius JG, Petersen BE (2002). In vitro trans-differentiation of adult hepatic stem cells into pancreatic endocrine hormone-producing cells. Proc Natl Acad Sci USA.

[54] Ende N, Chen R, Reddi AS (2004). Effect of human umbilical cord blood cells on glycemia and insulitis in type 1 diabetic mice. Biochem Biophys Res Commun.

[55] Oh SH, Muzzonigro TM, Bae SH, LaPlante JM, Hatch HM, Petersen BE (2004). Adult bone marrow-derived cells trans-differentiating into insulin-producing cells for the treatment of type I diabetes. Lab Invest.

[56] Hori Y, Gu X, Xie X, Kim SK (2005). Differentiation of insulin producing cells from human neural progenitor cells. PLoS Med.

[57] Chao KC, Chao KF, Fu YS, Liu SH (2008). Islet-like clusters derived from mesenchymal stem cells in Wharton’s Jelly of the human umbilical cordfor transplantation to control type 1 diabetes. PloS One.

[58] Koblas T, Harman SM, Saudek F (2005). The application of umbilical cord blood cells in the treatment of diabetes mellitus. Rev Diabet Stud.

[59] Rahman A, Isenberg DA (2008). Systemic lupus erythematosus. N Engl J Med.

[60] Sun L, Wang D, Liang J, Zhang H, Feng X, Wang H (2010). Umbilical cord mesenchymal stem cell transplantation in severe and refractory systemic lupus erythematosus. Arthritis Rheum.

[61] Majithia V, Geraci SA (2007). Rheumatoid arthritis: diagnosis and management. Am J Med.

[62] Scott DL, Wolfe F, Huizinga TW (2010). Rheumatoid arthritis. Lancet.

[63] Liu Y, Mu R, Wang S, Long L, Liu X, Li R (2010). Therapeutic potential of human umbilical cord mesenchymal stem cells in the treatment of rheumatoid arthritis. Arthritis Res Ther.

[64] Finckh A, Ciurea A, Brulhart L, Kyburz D, Möller B, Dehler S (2007). B cell depletion may be more effective than switching to an alternative anti-tumor necrosis factor agent in rheumatoid arthritis patients with inadequate response to anti-tumor necrosis factor agents. Arthritis Rheum.

[65] Atiyeh BS, Gunn SW, Hayek SN (2005). State of the art in burn treatment. World J Surg.

[66] Luo G, Cheng W, He W, Wang X, Tan J, Fitzgerald M (2010). Promotion of cutaneous wound healing by local application of mesenchymal stem cells derived fromhuman umbilical cord blood. Wound Repair Regen.

